# GWAS Reveals Key Candidate Genes Associated with Milk-Production in Saanen Goats

**DOI:** 10.3390/ani15223282

**Published:** 2025-11-13

**Authors:** Fu Li, Yonglong He, Hanbing Yan, Jiaqi Bu, Zhanhang Wang, Xiaolong Xu, Danni Li, Binyun Cao, Xiaopeng An

**Affiliations:** College of Animal Science and Technology, Northwest A&F University, Yangling 712100, China; lifudky@163.com (F.L.); heyonglong96@163.com (Y.H.); yanhanbing1128@163.com (H.Y.); 15598615360@163.com (J.B.); wzh1450448088@163.com (Z.W.); xuxiaolongxs@163.com (X.X.); lidanni@nwafu.edu.cn (D.L.); caobinyun@126.com (B.C.)

**Keywords:** GWAS, dairy goat, milk-production, candidate genes, SNP

## Abstract

Milk-production is one of the most important economic traits in dairy goats. Fundamentally, increasing milk-production at the genetic level can provide potential genetic markers for improving milk-production in dairy goats. Previous studies have shown that milk-production traits in dairy goats are highly polygenic and can be influenced by multiple genes. We therefore performed high-throughput sequencing on 350 Saanen dairy goats and conducted a genome-wide association study (GWAS) on the sequencing and production data. A total of 9,667,930 valid SNPs were identified, among which approximately 51% of the mutations were synonymous changes, and apart from unknown mutations, only a few changes affected gene expression. Finally, 318 SNP loci and 244 candidate genes were selected. We selected 10 loci most likely to influence these traits for large-scale population-based identification. Finally, four candidate genes, *CDC14A*, *F11*, *RBPJL* and *ZFAND2A*, were identified as the most likely to affect milk-production traits in dairy goats. To prevent spurious association analyses, we conducted subsequent experiments. Further results showed that the four candidate genes could regulate the proliferation and secretion of dairy goat mammary epithelial cells by regulating MAPK, mTOR and other pathways. These findings provide more valuable genetic markers and a theoretical basis for better understanding the intrinsic mechanisms of dairy goat mammary glands.

## Simple Summary

Milk-production is crucial for dairy goats’ value. We sought genes affecting this by studying 350 Saanen goats. We found four genes linked to milk-production, working through certain pathways. Breeders can use this research to boost yields. Scientists gain knowledge on mammary function, aiding dairy research and industry.

## 1. Introduction

Milk-production, which is deemed one of the most significant economic traits in dairy goats [1], is influenced by external environmental factors, genetics, nutritional status, and other variables [2,3]. Milk is secreted by mammary epithelial cells, passes through various mammary ducts into the mammary cistern, and exits via the nipple. The lactation period of dairy goats lasts 7–9 months and is typically divided into early, mid, peak, and late stages of milk-production. Several studies have demonstrated that milk-production is regulated by multiple genes related to lactation, epigenetic regulatory genes, and other regulatory molecules such as mRNA and circular RNA (circRNA), with production increasing exponentially during the lactation cycle [4,5]. Molecular breeding technology have been applied to select and breed Xinongsaanen goats, significantly shortening the generation interval and reducing unnecessary labor [6,7].

A genome-wide association study (GWAS) aims to obtain genotypes by detecting polymorphisms of genome-wide genetic variants (markers) across multiple individuals, followed by statistical analysis at the population level using observable traits, namely phenotypes [8]. Markers, namely insertion–deletion (indel) or single-nucleotide polymorphisms (SNPs) variations, are most likely to influence traits, are selected based on statistical significance or *p*-values to elucidate the complex relationship between genotype and phenotype across different species [9]. Through GWAS, variant SNPs can be identified within plant and animal genomes, allowing for the identification of key genes involved in regulating important host economic traits [10,11]. Tan et al. constructed a comprehensive map using weighted gene co-expression network analysis (WGCNA), integrating typical genomic variant SNPs and transcriptional signatures related to muscle development. They proposed a novel regulatory target, namely the SOX6-MYH1s axis, governing breast muscle production and myopathy [12]. The high abundance of SNPs, reduced linkage disequilibrium, and presence of diverse subpopulations make GWAS an ideal breeding tool for exploring milk-producing candidate genes and molecular regulatory mechanisms underlying lactation output of dairy goat breeds, which is vital for advancing animal husbandry [13].

Although prior research has illuminated the genetic underpinnings of milk-production traits in dairy species [14,15,16], a comprehensive understanding of the specific genes and biological pathways underlying milk-production traits in Saanen goats remains incomplete. Given the economic importance of milk-production and the need for sustainable genetic improvement in this breed, there is an urgent need to identify key candidate genes using GWAS. The present research sought to bridge this scientific gap through GWAS analysis in a large population of Saanen goats, with the goal of uncovering novel genetic variants and candidate genes that significantly influence milk-production. The findings are expected to advance the comprehension of molecular regulatory mechanisms regulating lactation in goats and provide valuable genetic markers for marker-assisted selection (MAS) and genomic selection (GS) programs [17], ultimately facilitating the breeding of high-yielding Saanen goats with enhanced productivity and economic efficiency.

## 2. Materials and Methods

The experimental protocol was approved by the Ethics Committee of Northwest A&F University. The experiments were conducted in strict accordance with the “Management Measures for Laboratory Animals of Northwest A&F University” and the “Guidelines for Welfare and Ethics Review of Laboratory Animals” (GB/T 35892-2018).

### 2.1. Sample Collection

In this experiment, 350 Saanen dairy goats with milk-production of more than 5 kg and less than 3 kg were collected from Shaanxi Province, China. A total of 200 Saanen dairy goats with milk-production within the normal range (3 kg < milk-production < 5 kg) were sampled from Gansu Province. The discovery cohort (*n* = 350) and validation cohort (*n* = 200) were derived from two geographically separated farms with no shared ancestry, ensuring independence. Notably, the discovery cohort (*n* = 350) comprises extreme phenotypic groups, specifically 150 goats with high milk yield (>5 kg/day) and 200 goats with low milk yield (<3 kg/day). The validation cohort (*n* = 200) is an independent population with average milk yield (3–5 kg/day). Goats were of the same age, without disease, and had similar body conditions. All goats were in the middle of the second lactation period. The milk-production measurement begins 15 days after the birth of the lamb (to exclude colostrum) and ends 280 days after the birth (this is the average lactation period of Saanen goats). The daily average milk-production is calculated by dividing the total milk-production (the sum of daily milk yields) from 15 days after the birth to 280 days by 265 days (the effective lactation days). Blood samples (5 mL) were drawn from a negative pressure collection vein for DNA extraction. The average milk-production of dairy goats during the second lactation period was recorded every three days. Finally, the average of all recorded values was used as the dairy goat phenotype record.

Goat whole blood DNA was extracted and purified using a whole blood/tissue/cell Genomic DNA Rapid Extraction Kit (DN1002; Aidlab Biotechnologies Co. Ltd., Beijing, China). The extracted DNA samples were analyzed using the following methods: (1) DNA purity was assessed by 1% agarose gel electrophoresis, and (2) DNA concentration was accurately quantified using a Qubit fluorometer. Samples with OD values between 1.75 and 1.85 and concentrations exceeding 100 μg/mL were selected and stored at −80 °C for future use.

### 2.2. Resequencing of the Whole Genome

Whole-blood DNA was extracted from 350 milk-yield-recorded samples, with quality verified by agarose gel electrophoresis and Qubit. For genotyping, 50 individuals underwent 10× average depth whole-genome resequencing (WGS) to build an imputation panel, while the remaining 300 were sequenced at 1× depth for imputation. All samples were sequenced on the same Illumina NovaSeq 6000 PE150 platform (Illumina, San Diego, CA, USA) [18]. Raw reads were quality-controlled via FASTP (v0.20.0; parameters: -n 10 -q 20 -u 40) [19], then aligned to the GCF_001704415.1_ARS1 reference genome using BWA-MEM [20]. For the imputation panel, GATK [21] HaplotypeCaller processed 50 WGS bam files to generate SNPs, followed by Beagle v5.4 for haplotype panel construction [22]. Loimpute [23] was used to impute 300 low-coverage samples. Merged genotypes of 350 individuals were filtered with PLINK [24] (parameters: -maf 0.05 -mind 0.1 -geno 0.1) for subsequent GWAS.

### 2.3. Genome-Wide Association Study

Following quality control of mutation results, population genetic analysis was conducted to calculate the parameters required for the GWAS model in five steps: phylogenetic tree construction, population structure analysis, principal component analysis, kinship analysis, and LD decay analysis. Based on the SNP markers obtained after screening, a NJ tree was constructed using the MEGA-X software (X 10.2.6) (model: p-distance; bootstrap: 1000 iterations) and the population structure was inferred using the Admixture software (version 1.3). The Q matrix was derived from the population structure matrix at the optimal K value of admixture, while the K matrix was the affinity matrix calculated by Genome-wide Complex Trait Analysis (GCTA). At the same time, using the GCTA software (1.93.3beta2), a PCA analysis was conducted based on the filtered SNP markers. The software Haploview 4.2 was used to calculate the LD size (r^2^) between each pair of markers, and the changes in this value as the distance increased were plotted. In this study, GWAS was performed using Tassel with the common model. The overall genome-wide association analysis model was as follows:

Y = Xα + Qβ + kμ + e

where Y is the phenotype vector, X is the genotype matrix, α vector is the genotype effect, Q is the fixed effect matrix (used for population structure/sex/location/account and other information), β vector is the fixed effect vector, k is the random effect matrix (mainly referring to the genetic relationship matrix), μ vector is the random vector effect, and e is the residual vector effect.

Manhattan and Q-Q plots were generated by transforming *p*-values with −log10 for visualization. A significance threshold of 5 × 10^−8^ was selected, which is commonly used in the article. The SNPs were annotated using the SnpEff software 5.1 [25] to determine their positions in the gene elements, the effects on amino acids, and other aspects.

### 2.4. Go Enrichment and Kegg Pathway Analysis

First, candidate genes for each GO term were filtered by mapping them to a database (http://www.geneontology.org/) [26] and calculating the count of genes linked to each term. This process yielded a list of genes corresponding to the three primary GO categories—molecular function, cellular component, and biological process Hypergeometric tests were then performed to identify GO terms significant enrichment of candidate genes was observed versus the whole-genome baseline. A 0.05 corrected *p*-value threshold was used to identify significantly enriched GO terms.

In addition to GO analysis, the KEGG database (http://www.genome.jp/kegg/) (accessed on 6 September 2021) [27] analysis of large-scale molecular datasets was utilized to further decipher the biological function of the candidate genes. A corrected *p*-value of 0.05 was also used to identify significantly enriched KEGG pathways.

### 2.5. Correlation Analysis

SNPs were genotyped using PCR followed by sequencing (Table 1). The PCR products were sent to the Sangon Biotech Shaanxi Sequencing Department (Shaanxi, China) for sequencing. Results were analyzed using SnapGene software (6.0.0), and nucleotide variations were annotated following the Human Genome Variation Society (HGVS) guidelines nomenclature guidelines (http://www.HGVS.org/varnomen) (accessed on 27 September 2021). Genotype, allele frequency, polymorphism information content (PIC), heterozygosity (He) and were calculated for each SNP. Harden-Weinberg equilibrium was assessed using *p*-value calculations, and a Chi-square (χ^2^) test was performed.

### 2.6. Cell Culture and Transfection

Goat mammary epithelial cells (GMECs) were isolated from mammary tissue of primary dairy goats collected at the peak of lactation and cultivated in DMEM/F-12 medium (Cytiva, SH30272.01 Uppsala, Sweden). Then, 1% pen/strep (Thermo Fisher, 15140122 Waltham, MA, USA) was added as a supplement and 10% fetal bovine serum (ZETA LIFE, Z7185FBS-500 Shenzhen, China). The GMECs were transfected with plasmid (1 μg/μL) for 24 h using LIPOFECTAMINE^®^ 2000 Reagent (Thermo Fisher Scientific, 2270659). The overexpression plasmids for CDC14A (goat, gene ID: 102176004), F11 (goat, gene ID: 102184496), RBPJL (goat, gene ID: 102186030), and ZFAND2A (goat, gene ID: 102171397) were engineered by Sangon Biotech (Shanghai) Co., Ltd. Shanghai, China. The siRNAs for the four genes were engineered by Shanghai Gima Pharmaceutical Technology Co., Ltd. Shanghai, China (Supplementary Materials). Transfection was performed following the Lipofectamine™ RNAiMAX Reagent (Invitrogen, 13778030, Carlsbad, CA, USA) transfection reagent instructions. After culturing the GMECs in 6-well plates (4−6 × 10^6^ cells/well), NC (50 nM), si-CDC14A (50 nM), si-RBPJL (50 nM), and si-F11 (50 nM) were mixed with 5 μL RNAiMAX up to a final volume of 200 μL. Then, the Opti-MEMI medium was transfected into the cells (*n* = 6). According to the Lipofectamine™ 3000 (Invitrogen, 13778030) reagent experimental protocol, transfections were performed using Opti-MEM™ culture based dilute release Lipofectamine™ 3000 test agent. Plasmids, including pcDNA3.1 (4 μg), and pcDNA3.1-CDC14A (4 μg), pcDNA3.1-RBPJL (4 μg), pcDNA3.1-F11 (4 μg), and pcDNA3.1-ZFAND2A (4 μg), were diluted in Opti-MEM™ to prepare the DNA premix. The P3000™ reagent was then added, and the diluted DNA was mixed (1:1) with the diluted Lipofectamine™ 3000 reagent. After a 15 min incubation, the DNA-lipid complexes were added to the cells (*n* = 6). The amount of reagent used was halved sequentially for the 12-, 24-, 48-, and 96-well plates (*n* = 6). Subsequent experiments on the cells were conducted 24 h after transfection.

### 2.7. Rt-Qpcr

Total cellular RNA was extracted using the SevenFast^®^ Total RNA Extraction Kit for cells (Seven Innovation Biological Technology Co., Ltd., Beijing, China, 24HM0023). cDNA synthesis was performed using Takara’s PrimeScript™ RT Kit with gDNA Eraser (RR047A). RT-q PCR was performed using SYBR Premix Ex Taq (Takara, RR820A, Shiga, Japan) and the primers listed in Supplementary Materials. The expression level of β-actin was used as an internal control, while the fold change in mRNA was determined via the 2^−ΔΔCt^ algorithm using Ct values.

### 2.8. Cell Proliferation Assay

Cell viability was determined using the AR Cell Proliferation and Toxicity Detection Kit (CCK8) (AccuRef Scientific, AC0011, Xi’an, China). A total of 100 µL of cell suspension was dispensed into each 96-well plate well, and the culture plates were pre-cultured at 37 °C with 5% CO_2_ for 24, 48, 72 and 96 h. Then, 10 µL CCK8 solution was dispensed into each well. The culture plates were then incubated for an additional 2–4 h, and absorbance at 450 nm was quantified with a Biotek Epoch microplate reader (Biotek, Winooski, VT, USA).

### 2.9. Edu Assay

An EdU Apollo567 in vitro kit (RiboBio, C10310-1, Guangzhou, China) was used to assess the proliferation of cultured cells in vitro. Firstly, the cells were first labeled with a pre-diluted EdU solution. After fixation with cell fixative, Apollo staining and DNA staining were performed. Lastly, the slides were cover slipped using an anti-fade mounting medium and examined via fluorescence microscopy.

### 2.10. Annexin-V Staining

Annexin V staining was performed as previously described [28]. Following transfection with overexpression plasmids or siRNA, cells were collected and resuspended in 150 µL of binding buffer. The cell suspension was stained with Annexin V-FITC/PI for 15 min in the dark and subsequently analyzed by flow cytometry (BD Canto II, BD Biosciences, Piscataway, NJ, USA).

### 2.11. Statistical Analysis

Results from at least three independent experiments were compiled. Data are presented as the mean ± SEM. We have included age at kidding, number of lactations, and feeding conditions as fixed effects in the GWAS model. SnapGene software 3.2.1 was used to view SNPs in the gene sequences of the samples. Excel was used to compute allelic frequencies, He, and PIC. Chi-square (χ^2^) and *p* values based on the Hardy–Weinberg equilibrium was calculated using Excel. Statistical analysis was performed using univariate analysis via one-way ANOVA in SPSS 18 and *p* < 0.05 (*) or *p* < 0.01 (**) was considered statistically significant. When the normality assumption was not satisfied, the Kruskal–Wallis test was applied. Graphs were generated using GraphPad Prism software v6.01.

## 3. Results

### 3.1. Overview of Sequencing Data

**Figure 1:**
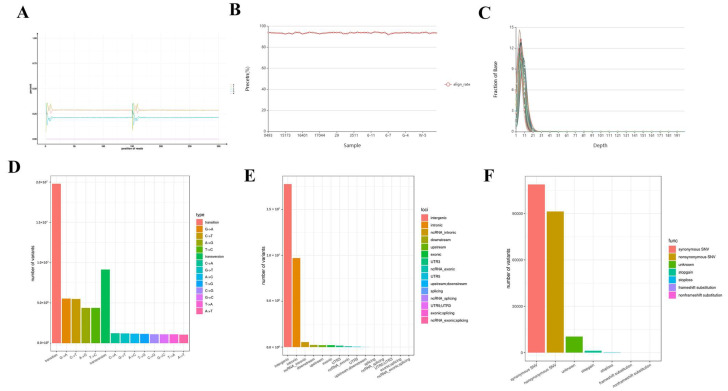
Summary of Sequencing Analysis Data. (**A**) Example of base type (A, T, C, G) distribution in a single sequencing sample. (**B**) Statistical plot of alignment results between samples and reference genomes. (**C**) Sequencing depth coverage plot. (**D**) Statistical plot of SNP types (e.g., transitions: A→G, G→A, C→T, T→C; transversions: A→C, A→T, C→A, G→T, G→C, T→A, T→C). (**E**) Statistical plot of SNP position annotation (e.g., intergenic, intronic, exonic). (**F**) Statistical plot of SNP functional annotation (e.g., synonymous SNV, nonsynonymous SNV, stopgain).

All sequencing data have been uploaded to the SRA database by the NCBI, with the accession numbers PRJNA904518 and PRJNA1230755. A total of 10 billion raw reads were generated. On average, each sample yielded approximately 27 Gb of sequence data, approximately nine times the size of the 2.8 Gb dairy goat genome. Following the removal of adapter sequences, low-quality reads, and reads containing > 10% ambiguous bases, (Ns) across the read length or over 40% low-quality bases, approximately 9 billion clean reads remained, corresponding to an average of 22 Gb per sample. This was followed by comparison to the reference genome GCF_001704415.1_ARS1. The base composition of A, T, C, and G was consistent across samples, with no AT and CG segregation and an approximately uniform distribution across the genome (Figure 1A). Comparison with the reference genome indicated high similarity between the sequencing data and the reference (Figure 1B). The sequencing depth coverage plot further confirmed the reliability and quality of the sequencing data (Figure 1C).

### 3.2. Comparison of Reference Genome Maps

SNPs were identified by aligning clean reads to the reference genome using the Unified Genotyper module in GATK software (version v3.5-0-g36282e4). A total of 9,686,439 SNPs were detected. The frequency of conversion was significantly higher than that of other variants, with approximately two-thirds of the SNPs and other variants containing conversion variants (Figure 1D). Functional annotation of identified genetic variants was conducted using ANNOVAR 20200608. Based on genomic location (Figure 1E), the sequences mapped onto the genome were classified into intergenic, intronic, exonic, and noncoding regions. Given that SNPs in exonic regions can affect protein translation, these were further annotated (Figure 1F). The results showed that approximately 51% of the exonic mutations were synonymous. Aside from a small number of mutations with unknown effects, only a few were predicted to impact gene expression.

### 3.3. Identification of Snp Mutations

**Figure 2:**
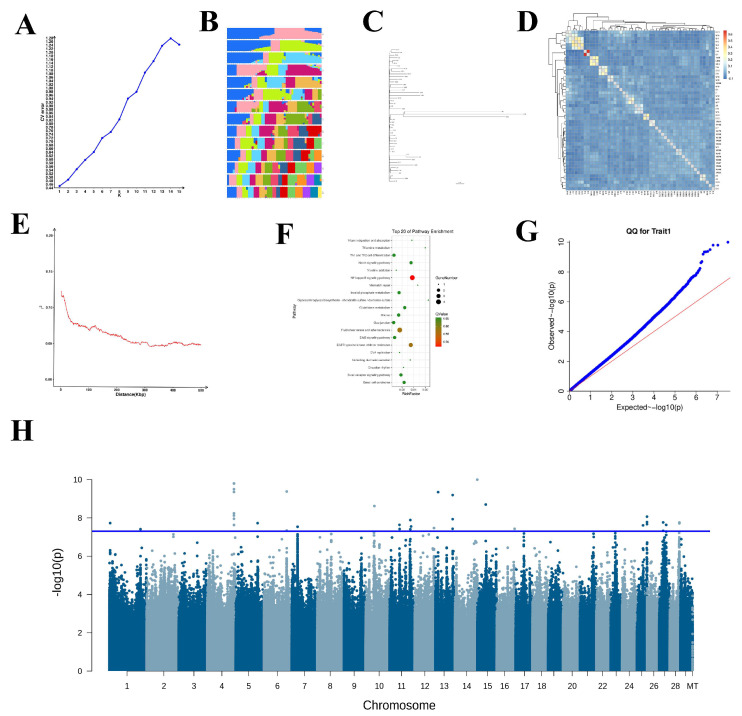
Summary of Sequencing Analysis Data. (**A**). Line plot of cross-validation error rates for population structure analysis (e.g., K = 1 to K = 10). (**B**) Bar graph of genetic composition of samples from population structure analysis. (**C**) Phylogenetic tree of evolutionary relationships among populations. (**D**) Sample relatedness matrix construction. (**E**) Linkage disequilibrium (LD) decay plot (r^2^). (**F**) KO (Kyoto Encyclopedia of Genes and Genomes) enrichment bubble plot. (**G**) Q–Q (quantile–quantile) plot. (**H**) Manhattan plot of genome-wide association studies (GWAS).

A total of 9,667,930 high-quality SNPs were retained following the filtration of loci with minor allele frequency (MAF) < 0.05 and a deletion rate exceeding 5%. The population structure effect matrix was calculated, and population analyses were conducted (Figure 2A–E). Subsequently, GWAS was conducted to screen for SNP loci significantly associated with milk-production traits in dairy goats. In total, 318 SNPs reached statistical significance, with 64% (202 SNPs) located in intergenic regions, 31% (98 SNPs) in intronic regions, and only 5% (18 SNPs) in exons or noncoding regions. including upstream and downstream regions. After re-analysis with the 5e-8 threshold, 38 SNPs remained significantly associated with milk yield (reduced from 318 SNPs with the previous threshold) (Table 2). From these, 244 significant candidate genes were identified and annotated based on the physical positions of their corresponding SNPs on the chromosomes (Figure 2G,H).

### 3.4. Go and Kegg Analysis

In the present study, 318 significant SNP sites were annotated within 244 candidate genes, indicating that some sequences contained multiple mutation sites. For example, 26 SNPs were identified between LOC108633170 and LOC102170513, Five SNPs were mapped to the exonic and intronic regions of the F11 gene, and 12 SNPs were found in the upstream to downstream regions of PCNX2. Since base mutations can influence the expression of candidate genes, the GO database was used to analyze these SNP-containing candidate genes. In total, 244 candidate genes corresponding to the 318 SNPs were annotated with 86 GO terms. Focusing on terms with *p* < 0.05, eight significant GO terms were identified (Table 2). In the cell component category, three GO terms—cilium (GO: 0005929), cell projection (GO: 0042995), and plasma membrane-bound cell projection (GO: 0120025)—were significantly enriched. Only cell division (GO:0051301) showed significant enrichment in the biological process category.

KEGG pathway enrichment analysis was conducted to further investigate the biological functions of these SNP-associated candidate genes. The annotation revealed that 178 candidate genes were associated with 151 biological processes. Moreover, eight KEGG pathways were significantly enriched with 23 candidate genes (*p* < 0.05), indicating their potential key roles in dairy goat lactation (Table 3). Among these, the NF-kappa B signaling pathway (ko04064) is notably prominent (Figure 2F).

### 3.5. Validation of Snps Through Association Analysis

**Figure 3:**
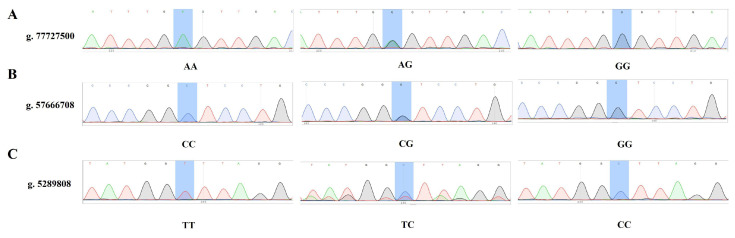
Peak Plots of Genotypes for Selected SNPs in Different Samples. (**A**) Peak plots of genotypes for the g. 77727500 SNP in different samples. (**B**) Peak plots of genotypes for the g. 57666708 SNP in different samples. (**C**) Peak plots of genotypes for the g. 5289808 SNP in different samples.

To verify whether the significant SNP loci identified through screening truly represent significantly mutated loci, association analysis was conducted to examine the correlation between these SNP loci and milk-production-related phenotypic traits. Ten SNP loci most likely to influence milk-production in dairy goats were selected, and external primers were designed accordingly. A random subset of 200 samples was chosen for locus sequencing validation via Sanger sequencing. Figure 3 presents a partial sequencing map, and mutant site genotypes were identified based on sequencing results. Allele frequencies for each locus were calculated, and PIC and He were assessed according to Hardy–Weinberg equilibrium (Table 4). The results indicated that loci g. 77727500 and g. 5289808 showed significant deviation from Hardy–Weinberg equilibrium (*p* < 0.05). Regarding PIC, loci g. 77727500, g. 5289808, g. 57666708, g. 73139883, g. 27027033, g. 37633188, g. 29255238 exhibited moderate polymorphism (0.25 < PIC < 0.5), whereas loci g. 39072994, g. 19470992, g. 42365731 showed low polymorphism (PIC < 0.25).

The correlation between genotypes at the 10 SNP loci and milk-production phenotypes was analyzed (Table 5 and Table 6). Results showed that the AA genotype at g. 77727500 had a significantly higher milk-production than that of the AG genotype. Similarly, the TT genotype at g. 73139883 and g. 27027033 had significantly higher milk-productions than those of the CT and CC genotypes. The AA genotype at g. 37633188 showed significantly higher milk-production than that of the CA and CC genotypes. The milk-production of the AA and GA genotypes at g. 19470992 was significantly higher than that of the GG genotype; the milk-production of the CA genotype at g. 42365731 was significantly higher than that of the CC genotype; and the milk-production of the CC genotype at g. 29255238 was significantly higher than that of the TC genotype, which was in turn significantly higher than that of the TT genotype. These findings suggest that candidate genes CDC14A, RBPJL, ZSCAN9, ZFAND2A, and F11 may influence lactation in dairy goats.

### 3.6. Effect of Overexpressing Candidate Genes on the Lactation Performance of Gmecs

**Figure 4:**
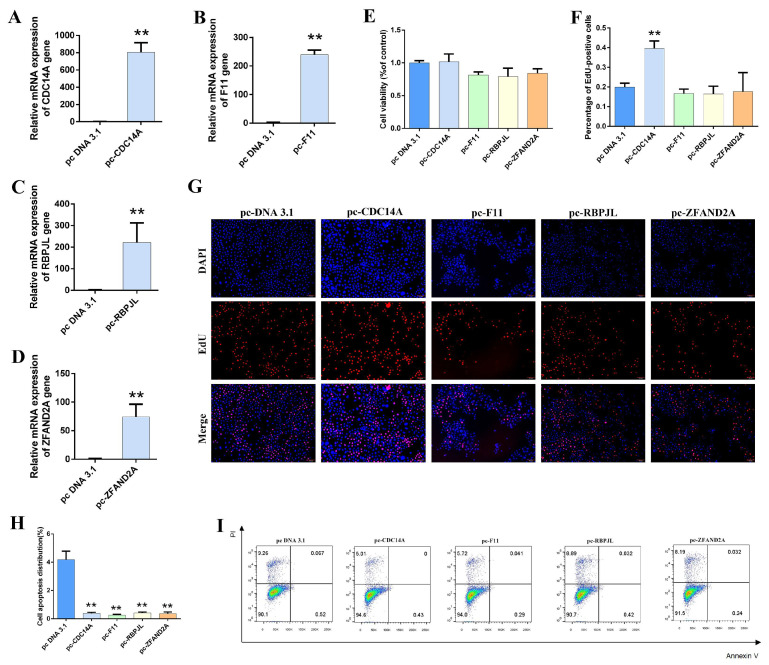
Functional Characterization of Candidate Gene Overexpression. (**A**–**D**) Efficiency assays of overexpression plasmids (*n* = 4). (**E**) GMECs (goat mammary epithelial cells) viability assay (*n* = 4). (**F**,**G**) GMECs proliferation assays (*n* = 6). (**H**,**I**) Assays of GMECs apoptosis rate (*n* = 6). Data are presented as mean ± SEM. ** *p* < 0.01 vs. NC group.

Based on the above results, to further explore the regulatory roles of candidate genes in GMEC proliferation and apoptosis and lactation performance, we constructed overexpression vectors for four candidate genes: pcDNA3.1-CDC14A (pc-CDC14A), pcDNA3.1-F11 (pc-F11), pcDNA3.1-RBPJL (pc-RBPJL), and pcDNA3.1-ZFAND2A (pc-ZFAND2A; the ZSCAN9 gene was not verified due to lack of an accurate sequence). Once the gene sequence is determined, we will proceed to conduct further validation of the function of this gene). GMECs were seeded in six-well plates and, once reaching the logarithmic growth phase, transfected with the overexpression vectors of candidate genes or the empty pcDNA3.1 vector. RNA was extracted 24 h post-transfection and reverse-transcribed for RT-qPCR analysis to assess transfection efficiency. The results showed a significant increase in mRNA levels for all candidate genes (Figure 4A–D), confirming the effectiveness of the overexpression vectors. Overexpression of CDC14A significantly inhibited cell viability and proliferation, whereas overexpression of the other candidate genes had no significant effect on these parameters (Figure 4E–G), indicating that CDC14A play a critical role in the regulation of cell proliferation. Apoptotic cell counts were quantified by Annexin V-FITC/PI staining followed by flow cytometric analysis, revealing that all candidate gene overexpression groups exhibited significantly fewer apoptotic cells compared to the blank control, suggesting that these genes inhibit apoptosis (Figure 4H,I). Furthermore, analysis of lactation-related genes showed that the overexpression of candidate genes significantly regulated the expression of key genes in milk fat biosynthesis (FASN, SREBP1, PPARG, and FABP3) and milk protein synthesis (CSN2, CSN3, and CSN1S2), as well as genes associated with the Janus kinase-signal transducer and activator of transcription (JAK-STAT) and mammalian target of rapamycin (mTOR) signaling pathways (Supplementary Materials).

### 3.7. Effect of Silent Candidate Genes on the Lactation Performance of Gmecs

**Figure 5:**
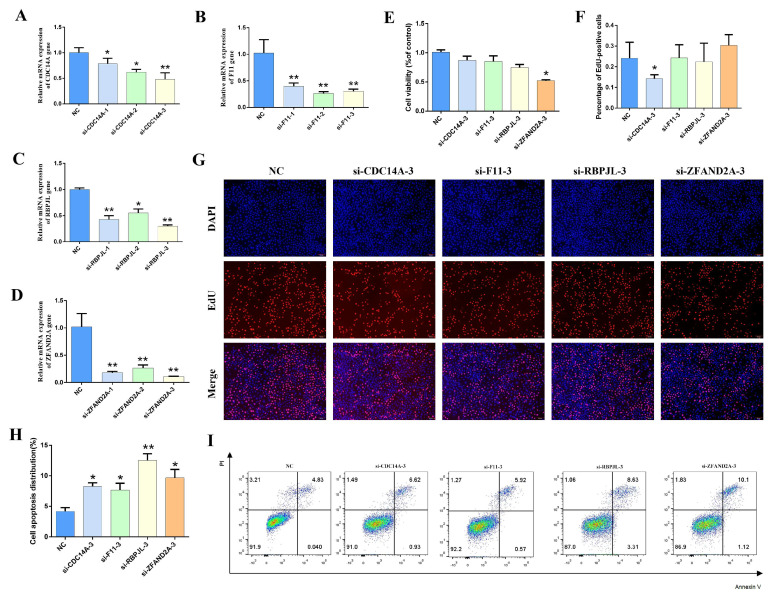
Functional Characterization of Candidate Gene Silencing. (**A**–**D**) Efficiency assays of siRNA (small interfering RNA)-mediated gene silencing (*n* = 4). (**E**) GMECs viability assay (*n* = 4). (**F**,**G**) GMECs proliferation assays (*n* = 6). (**H**,**I**) Assays of GMECs apoptosis rate (*n* = 6). Data are presented as mean ± SEM. * *p* < 0.05, ** *p* < 0.01 vs. NC group.

To comprehensively verify the effects of the candidate genes on cell proliferation and apoptosis, three pairs of interfering RNAs (siRNAs) were designed for each candidate gene sequence that matched the overexpression vector sequences. Gene silencing efficiency tests were performed to identify the most effective siRNAs for each gene (Figure 5A–D). Lipofectamine™ 3000 was used for siRNA gene knockdown experiments. The CCK8 assay showed that silencing ZFAND2A reduced cell viability, while silencing the other genes had no effect on cell viability (Figure 5E). As depicted in Figure 5F,G, silencing the CDC14A gene significantly decreased the number of EdU-positive cells compared to the negative control (NC) group, whereas silencing the other genes did not affect cell proliferation. Apoptosis assays revealed that silencing all four candidate genes promoted cell apoptosis (Figure 5H,I). Additionally, gene silencing regulated the expression of genes involved in milk fat and protein biosynthesis, and the JAK-STAT and mTOR signaling pathways to varying extents (Supplementary Materials).

## 4. Discussion

GWASs have become a key tool for functional gene mining owing to their high correlation accuracy and short research cycle [29]. With technological advancements, high-throughput sequencing has become a primary method for constructing high-density genetic marker panels [30,31]. By integrating high-throughput sequencing technology with GWAS, numerous genetic variants linked to complex traits have been identified [32,33,34]. Over recent years, this method has been extensively used to screen and identify major genes associated with economically important traits in both agricultural plants and animals [35,36,37], significantly advancing agricultural genetics. Furthermore, advances in sequencing technology have facilitated the in-depth study of genes and proteins in living organisms. Song et al. identified differentially expressed circRNAs during the transition from the pre-receptive to the receptive phase, demonstrating high stage specificity [26]. Marx et al. conducted proteomic sequencing of differential proteins in rhizobia from the leguminous plant alfalfa and its nitrogen-fixing endosymbionts [38]. In contrast, studies detecting SNPs related to milk-production and lambing in dairy goats remain relatively scarce. In this study, we generated 27 Gb of whole-genome resequencing data per sample, approximately nine times the size of the domestic goat genome (~2.88 Gb). After filtering out low-quality sequences, we obtained approximately 10 billion clean reads, providing sufficient sequencing depth to detect genome-wide genetic variation associated with milk-production traits. Compared to goats, humans exhibit a higher number of SNP mutations [39], which may be attributed to the slightly larger human genome (~3 Gb in humans vs. 2.92 Gb in goats) and the greater number of annotated genes. Nevertheless, of the goat genome also contains a large number of SNPs [40], and different gene mutations can be detected depending on the traits being studied [41,42]. Based on the mutation type, the impact of these SNPs on specific goat traits could be determined. Notably, different target traits may be associated with the same gene mutations [43,44].

Compared to the reference genome, 9,667,930 SNPs were identified. The Manhattan plot from the GWAS revealed 318 significant SNPs. GO enrichment and KEGG pathway analyses were conducted on the candidate genes identified through GWAS, as these tools help researchers better understand gene expression products and their interactions [16]. The top three significantly enriched GO terms were cilium (GO:0005929), cell projection (GO:0042995), and plasma membrane-bound cell projection (GO:0120025), which may be of interest in future studies. These terms were enriched for four candidate genes, namely CDC14A, DISC1, DNAH3, and PKHD1, which have previously been reported to participate in biological processes such as intracellular material transport, DNA break repair, and testicular development [45,46]. Therefore, we postulate that these genes might also participate in breast cell proliferation and differentiation. In addition, a significantly enriched GO term related to molecular function, ATPase activity (GO:0016887), may indicate an energy-providing role in cells, consistent with previous findings. Our KEGG pathway analysis identified the NF-kappa B signaling pathway as highly significant. The NF-κB transcription factor is a key regulator of immune function, stress responses, apoptosis, and cell differentiation [47,48]. Genes such as TAB2, PLCG2, and PRKCQ may contribute to mammary gland development via this pathway, supporting the findings of our GO analysis.

SNP mutations in genes can disrupt the physiological processes of gene replication, transcription, and translation, potentially altering gene function [49]. Association analysis revealed that 7 of the 10 SNPs most likely to influence milk-production traits in dairy goats were significantly associated with these traits. Two loci showed a deviation from the Hardy–Weinberg equilibrium. The possible reasons are as follows: 1. Due to artificial selection, individuals do not have the same opportunities for survival and reproduction, and individual mating is not random, resulting in changes in gene frequencies. 2. Alleles can directly undergo mutations. 3. Due to artificial insemination, there may be the introduction of foreign genes, leading to changes in gene frequencies. 4. At the same time, the population we used for validating the loci is not large enough. Excluding unannotated genes, five candidate genes—CDC14A, F11, RBPJL, ZSCAN9, and ZFAND2A—were significantly associated with milk-production. Previous studies have shown that CDC14A, a phosphatase [50] is involved in cell mitosis, cell cycle regulation, and reproductive functions [46,51]. Consistent with these findings, our validation experiments demonstrated that CDC14A promoted GMEC proliferation and significantly inhibiting apoptosis. This suggests that its role in enhancing milk-production traits may be linked to its regulation of mammary epithelial cell proliferation. Our results confirmed that F11 also affects milk-production traits, influencing the synthesis of milk fat and protein. Along with the gene expression in the JAK-STAT pathway [52]. While F11 silencing did not significantly impact cell proliferation, silencing of all candidate genes (including F11) led to increased cell apoptosis. These findings suggest that this gene may regulate lactation in dairy goats via the neuroendocrine system. RBPJL is primarily expressed in the pancreas and participates in the growth, development, and function of pancreatic vesicles in animals [53], Because pancreatic function is closely related to feed digestion and nutrient absorption, it may indirectly influence lactation traits in dairy goats. A growing body of evidence has shown that RBPJL further contributes to cell differentiation [54], and its differential expression in mammary glands suggests a possible role in regulating mammary function and lactation by influencing mammary epithelial cell differentiation. Our results showed that RBPJL inhibited GMEC apoptosis and significantly upregulated the expression of milk fat biosynthesis genes including PPARG and FABP3, indicating that RBPJL may affect mammary fat secretion by modulating fat digestion and absorption through the pancreas. Zinc-finger proteins are recognized to be pivotal in stress regulation and resistance [55,56]. In this research, our focus was on ZFAND2A, as a definitive gene sequence for ZSCAN9 was unavailable. While ZFAND2A had no significant effect on GMEC proliferation, it was found to inhibit apoptosis. The JAK-STAT signaling pathway consists of a cascade of intracellular protein interactions involved in regulating immunity, cell division, apoptosis, and tumor development [57]. RT-qPCR results showed that silencing ZFAND2A significantly increased the expression levels of genes involved in milk fat biosynthesis and activated the JAK-STAT pathway, suggesting that ZFAND2A may inhibit milk fat secretion and apoptosis by suppressing JAK-STAT signaling.

In the global dairy goat industry, milk-production traits serve as the core determinant of farming profitability. The gene targets identified in this study can facilitate the breeding of high-yielding Saanen dairy goat strains. Meanwhile, the findings can be extrapolated to other breeds to enhance the overall quality and efficiency of the dairy goat industry. However, owing to the inherent limitations of GWAS, this methodology typically maps association signals to large chromosomal regions. Some significant association regions in this study may harbor dozens of candidate genes, necessitating integration with transcriptomic analyses or functional assays to further pinpoint the causal variants. The presence of inbreeding effects in the Saanen dairy goat population may introduce false-positive associations, warranting replication in independent populations (e.g., the Nordic Saanen dairy goat population). Additionally, functional validation of some genes (e.g., mTOR) in this study was confined to cell models (e.g., GMECs), whereas the absence of in vivo experiments (e.g., gene-knockout mice or goat models) to directly demonstrate their regulatory roles in milk-production traits may lead to functional annotation bias. Given that milk-production is a polygenic quantitative trait, the effect size of single candidate genes identified herein (e.g., STAT5) might be overestimated, and thus, the combined impact of gene–gene epistasis (e.g., the synergistic interaction between STAT5 and PPARG) should be considered in practical breeding programs.

## 5. Conclusions

In conclusion, our study identified candidate genes—CDC14A, F11, RBPJL, and ZFAND2A—as significantly associated with lactation performance in dairy goats, highlighting the important roles of the JAK-STAT and mTOR pathways in this process. Most importantly, this study provides evidence supporting the functions and pathways of these four genes in regulating lactation, which will inform future mechanistic and functional studies of these four candidate genes.

## Figures and Tables

**Table 1:** The information of ten SNPs.

SNP	Chromosome	Location	Mutations in the Former	After the Mutation	Candidate Genes
SNP-1	3	77,727,500	A	G	gene-*CDC14A*
SNP-2	5	5,289,808	T	C	gene-LOC108636124, gene-*PHLDA1*
SNP-3	10	57,666,708	C	G	gene-*ZNF609*
SNP-4	13	73,139,883	C	T	gene-*RBPJL*
SNP-5	20	39,072,994	G	A	gene-LOC102191110
SNP-6	5	27,027,033	C	T	gene-LOC102177517
SNP-7	15	37,633,188	C	A	gene-LOC102183585
SNP-8	23	19,470,992	G	A	gene-*ZSCAN9*
SNP-9	25	42,365,731	C	A	gene-*ZFAND2A*
SNP-10	27	29,255,238	T	C	gene-*F11*

**Table 2:** Significant SNP information.

Mark	Chr	Position	*p*
14_94113738	14	94,113,738	1.00 × 10^−10^
4_112826548	4	1.13 × 10^8^	1.58 × 10^−10^
4_112826558	4	1.13 × 10^8^	1.58 × 10^−10^
4_112828377	4	1.13 × 10^8^	3.16 × 10^−10^
6_96304798	6	96,304,798	4.17 × 10^−10^
4_112828332	4	1.13 × 10^8^	4.42 × 10^−10^
13_11635958	13	11,635,958	4.50 × 10^−10^
13_73078855	13	73,078,855	6.38 × 10^−10^
15_35470891	15	35,470,891	2.00 × 10^−9^
10_36720798	10	36,720,798	2.40 × 10^−9^
4_112824572	4	1.13 × 10^8^	5.77 × 10^−9^
4_112690246	4	1.13 × 10^8^	7.56 × 10^−9^
25_42375278	25	42,375,278	8.56 × 10^−9^
4_112819631	4	1.13 × 10^8^	1.10 × 10^−8^
13_73147747	13	73,147,747	1.16 × 10^−8^
11_87799604	11	87,799,604	1.30 × 10^−8^
25_42365217	25	42,365,217	1.64 × 10^−8^
27_16508513	27	16,508,513	1.72 × 10^−8^
28_40373888	28	40,373,888	1.74 × 10^−8^
1_3266310	1	3,266,310	1.86 × 10^−8^
5_92155728	5	92,155,728	1.87 × 10^−8^
28_39989356	28	39,989,356	1.92 × 10^−8^
25_42467165	25	42,467,165	2.12 × 10^−8^
11_41609340	11	41,609,340	2.30 × 10^−8^
27_29255284	27	29,255,284	2.31 × 10^−8^
4_112824616	4	1.13 × 10^8^	2.34 × 10^−8^
25_25701127	25	25,701,127	2.44 × 10^−8^
11_90960697	11	90,960,697	2.80 × 10^−8^
7_24603319	7	24,603,319	2.90 × 10^−8^
7_24613281	7	24,613,281	2.90 × 10^−8^
12_81575258	12	81,575,258	3.38 × 10^−8^
13_72876834	13	72,876,834	3.70 × 10^−8^
16_75562492	16	75,562,492	3.72 × 10^−8^
11_42215384	11	42,215,384	3.82 × 10^−8^
1_131580172	1	1.32 × 10^8^	3.90 × 10^−8^
11_87796475	11	87,796,475	3.93 × 10^−8^
6_96305737	6	96,305,737	4.57 × 10^−8^
27_16510017	27	16,510,017	4.74 × 10^−8^

**Table 3:** The important GO terms.

Ontology	GO ID	Description	Gene Ratio (30)	Bg Ratio (13,582)	*p* Value	Gene ID
Cellular Component	GO: 0005929	cilium	4	427	0.013822146	gene-*CDC14A*; gene-*DISC1*; gene-*DNAH3*; gene-*PKHD1*
Cellular Component	GO: 0042995	cell projection	4	427	0.013822146	gene-*CDC14A*; gene-*DISC1*; gene-*DNAH3*; gene-*PKHD1*
Cellular Component	GO: 0120025	plasma membrane bounded cell projection	4	427	0.013822146	gene-*CDC14A*; gene-*DISC1*; gene-*DNAH3*; gene-*PKHD1*
Biological Process	GO: 0051301	cell division	3	263	0.02431167	gene-*ACTR3*; gene-*ANK3*; gene-*MAP10*

**Table 4:** Pathways.

KEGG_A _Class	KEGG_B _Class	Pathway	chx (20)	All (8758)	*p* Value	Pathway ID	Genes	KOs
Environmental Information Processing	Signal transduction	NF-kappa B signaling pathway	4	103	0.0033965	ko04064	gene-*TAB2*; gene-*PLCG2*; gene-*PRKCQ*; gene-*CARD11*	K04404+K05859+K18052+K07367
Human Diseases	Cardiovascular disease	Fluid shear stress and atherosclerosis	4	143	0.010747	ko05418	gene-*BMP4*; gene-*PDGFA*; gene- *SDC4*; gene-*MGST1*	K04662+K04359+K16338+K00799
Human Diseases	Cancer: overview	Proteoglycans in cancer	3	209	0.011318	ko05205	gene-*SDC4*; gene-*PLCG2*; gene-*ANK3*	K16338+K05859+K10380
Human Diseases	Drug resistance: antineoplastic	EGFR tyrosine kinase inhibitor resistance	3	80	0.0123886	ko01521	gene-*PDGFA*; gene-*NRG1*; gene-*PLCG2*	K04359+K05455+K05859
Metabolism	Lipid metabolism	Steroid hormone biosynthesis	2	85	0.015792	ko00140	gene-LOC102188238; gene-LOC108633246	K00497+K00699
Organismal Systems	Development and regeneration	Axon guidance	4	179	0.0227066	ko04360	gene-*ROBO1*; gene-*PLCG2*; gene-*UNC5D*; gene-*PLXNA4*	K06753+K05859+K07521+K06820
Environmental Information Processing	Signal transduction	Notch signaling pathway	2	53	0.0406895	ko04330	gene-*RBPJL*; gene-*ATXN1*	K06053+K23616
Metabolism	Metabolism of cofactors and vitamins	Thiamine metabolism	1	20	0.0447426	ko00730	gene-*NTPCR*	K06928

**Table 5:** Genotypic distribution of SNP loci in candidate genes.

Locus			Frequency
g. 77727500	Genotype	AA (30)	0.14
		AG (79)	0.38
		GG (100)	0.48
	Allele	A	0.33
		G	0.67
	He		0.444
	PIC		0.345
	Equilibrium χ^2^ test		4.609
	*p*		0.032
g. 5289808	Genotype	TT (86)	0.41
		TC (74)	0.35
		CC (49)	0.24
	Allele	T	0.59
		C	0.41
	He		0.484
	PIC		0.367
	Equilibrium χ^2^ test		15.118
	*p*		0.0001
g. 57666708	Genotype	CC (118)	0.56
		CG (79)	0.38
		GG (12)	0.06
	Allele	C	0.75
		G	0.25
	He		0.371
	PIC		0.302
	Equilibrium χ^2^ test		0.066
	*p*		0.797
g. 73139883	Genotype	CC (98)	0.47
		CT (86)	0.41
		TT (25)	0.12
	Allele	C	0.67
		T	0.33
	He		0.439
	PIC		0.343
	Equilibrium χ^2^ test		0.821
	*p*		0.365
g. 39072994	Genotype	GG (191)	0.91
		GA (17)	0.08
		AA (1)	0.01
	Allele	G	0.95
		A	0.05
	He		0.087
	PIC		0.083
	Equilibrium χ^2^ test		0.821
	*p*		0.365
g. 27027033	Genotype	CC (104)	0.52
		CT (80)	0.40
		TT (15)	0.08
	Allele	C	0.72
		T	0.28
	He		0.400
	PIC		0.320
	Equilibrium χ^2^ test		0.005
	*p*		0.943
g. 37633188	Genotype	CC (42)	0.21
		CA (91)	0.455
		AA (67)	0.335
	Allele	C	0.44
		A	0.56
	He		0.492
	PIC		0.371
	Equilibrium χ^2^ test		1.142
	*p*		0.285
g. 19470992	Genotype	GG (142)	0.71
		GA (53)	0.265
		AA (5)	0.025
	Allele	G	0.84
		A	0.16
	He		0.265
	PIC		0.230
	Equilibrium χ^2^ test		0.0004
	*p*		0.984
g. 42365731	Genotype	CC (162)	0.82
		CA (34)	0.17
		AA (1)	0.01
	Allele	C	0.91
		A	0.09
	He		0.166
	PIC		0.152
	Equilibrium χ^2^ test		0.306
	*p*		0.580
g. 29255238	Genotype	TT (102)	0.51
		TC (78)	0.39
		CC (20)	0.10
	Allele	T	0.705
		C	0.295
	He		0.416
	PIC		0.329
	Equilibrium χ^2^ test		0.778
	*p*		0.378

**Table 6:** Association analysis of SNP loci with milk yield (means ± SEM).

Site	Genotype	Milk Yield (kg)
g. 77727500	AA (30)	3.98 ^a^ ± 0.20
	AG (79)	3.17 ^b^ ± 0.12
	GG (100)	3.28 ^ab^ ± 0.11
g. 5289808	TT (86)	3.40 ± 0.12
	TC (74)	3.45 ± 0.13
	CC (49)	3.03 ± 0.16
g. 57666708	CC (118)	3.17 ± 0.10
	CG (79)	3.52 ± 0.12
	GG (12)	3.83 ± 0.31
g. 73139883	CC (98)	3.06 ^b^ ± 0.10
	CT (86)	3.37 ^b^ ± 0.11
	TT (25)	4.30 ^a^ ± 0.20
g. 39072994	GG (191)	3.26 ± 0.07
	GA (17)	4.24 ± 0.46
	AA (1)	2.7
g. 27027033	CC (104)	3.18 ^b^ ± 0.10
	CT (80)	3.25 ^b^ ± 0.11
	TT (15)	4.69 ^a^ ± 0.25
g. 37633188	CC (42)	2.98 ^b^ ± 0.16
	CA (91)	3.14 ^b^ ± 0.11
	AA (67)	3.83 ^a^ ± 0.13
g. 19470992	GG (142)	3.09 ^b^ ± 0.09
	GA (53)	3.91 ^a^ ± 0.14
	AA (5)	4.01 ^a^ ± 0.46
g. 42365731	CC (162)	3.21 ^b^ ± 0.91
	CA (34)	3.92 ^a^ ± 1.60
	AA (1)	3.3 ^ab^
g. 29255238	TT (102)	3.03 ^c^ ± 0.10
	TC (78)	3.42 ^b^ ± 0.11
	CC (20)	4.59 ^a^ ± 0.22

^a–c^ Within a row, means with different superscripts differ significantly (*p* < 0.05).

## Data Availability

The datasets used and/or analyzed during the current study are available from the corresponding author on request.

## Supplementary Materials

The following supporting information can be downloaded at https://www.mdpi.com/article/10.3390/ani15223282/s1. Figures S1 and S2: mRNA Expression of Lactation-Related Genes; Tables S1–S2: Primer information.

## Abbreviations

The following abbreviations are used in this manuscript:

circRNA	circular RNA
GMECs	Goat mammary epithelial cells
GCTA	Genome-wide Complex Trait Analysis
GS	Genomic selection
GWAS	Genome-wide association study
He	Heterozygosity
HGVS	Human Genome Variation Society
indel	Insertion–deletion
MAS	Marker-assisted selection
MAF	Minor allele frequency
PIC	Polymorphism information content
SNPs	Single-nucleotide polymorphisms
WGCNA	Weighted gene co-expression network analysis
