# Supplementary material for: GWAS Reveals Key Candidate Genes Associated with Milk-Production in Saanen Goats
Source: Animals (Basel). 2025 Nov 13;15(22):3282. doi: 10.3390/ani15223282 (PMC12649104; doi:10.3390/ani15223282)
Supplement: Supplementary file 1 [file animals-15-03282-s001.zip › animals-3958058-supplementary.pdf]

Supplementary Materials

Table S1 Primer sequence of siRNA

siRNA	gene	sequence	
		sense（5'-3'）	antisense（5'-3'）
si-CDC14A-1	CDC14A	GCUGUAAACUAAACAAGAATT	UUCUUGUUUAGUUUACAGCTT
si-CDC14A-2	CDC14A	GCUUAUGCCGUAAUCUAUUTT	AAUAGAUUACGGCAUAAGCTT
si-CDC14A-3	CDC14A	GUGGAUGAAUAUGAACAUUTT	AAUGUUCAUAUUCAUCCACTT
si-F11-1	F11	GGAGCAAUUUCUGGAUAUUTT	AAUAUCCAGAAAUUGCUCCTT
si-F11-2	F11	GCACAAAGGAUCGUAACACTT	GUGUUACGAUCCUUUGUGCTT
si-F11-3	F11	GCUUGCAUUAGGGACAUUUTT	AAAUGUCCCUAAUGCAAGCTT
si-RBPJL-1	RBPJL	GCAAAGUAGCCAAACAGUGTT	CACUGUUUGGCUACUUUGCTT
si-RBPJL-2	RBPJL	GAGGCCGAAACCAUGUACATT	UGUACAUGGUUUCGGCCUCTT
si-RBPJL-3	RBPJL	GCCUUCUCCUUCACCUACATT	UGUAGGUGAAGGAGAAGGCTT
si-ZFAND2A-1	ZFAND2A	GGACAAAGUCGAUUAUGACTT	GUCAUAAUCGACUUUGUCCTT
si-ZFAND2A-2	ZFAND2A	GCACGUGGAUGGAGACUGUTT	ACAGUCUCCAUCCACGUGCTT
si-ZFAND2A-3	ZFAND2A	GGAGUCUCACUGUUUCUCUTT	AGAGAAACAGUGAGACUCCTT

Table S2 Primers information

SNP	Primer	Sequence (5'→3')	Product size (bp)	Tm (℃)
g. 77727500	F1	TCTACTGACCAGCACAATG	282	56
	R1	AGCGACCTAACCTACACT		
g. 5289808	F2	GGCTCATTATTCAAGGATACAG	298	52
	R2	GCAACCAGTGTTAAATGTCT		
g. 57666708	F3	CCGCTGACTTAGACATCTTA	424	56
	R3	GCTAACACAGAAGTTCCTCT		
g. 73139883	F4	CCCTGGCTGGTGTCTGTA	283	56
	R4	TTTATCCATTGCTTACCCT		
g. 39072994	F5	TGAAGGAAGGTAATAGGAAGG	276	56
	R5	TCGTGGATGTCTAACTTCTC		
g. 27027033	F6	CACCTTCGGAACCTTTAGC	173	55
	R6	CAGGGCAATCTCCTCGTAC		
g. 37633188	F7	AGTTTGGTCTGGAGGAGG	294	59
	R7	TGGAGCCATTCATAGTGT		
g. 19470992	F8	TAGCCTACAGACAGGAACA	279	55
	R8	TTGCTGGTAGAGCATTTT		
g. 42365731	F9	TGCTCACCTGTTACACCCTC	352	65
	R9	CTCGCCTGCCGTTACACT		
g. 29255238	F10	TTTTAGTTGGAGATCCATTTGCAGAAAGTCT	234	59
	R10	GCGGACGGGCATGAAGCC		

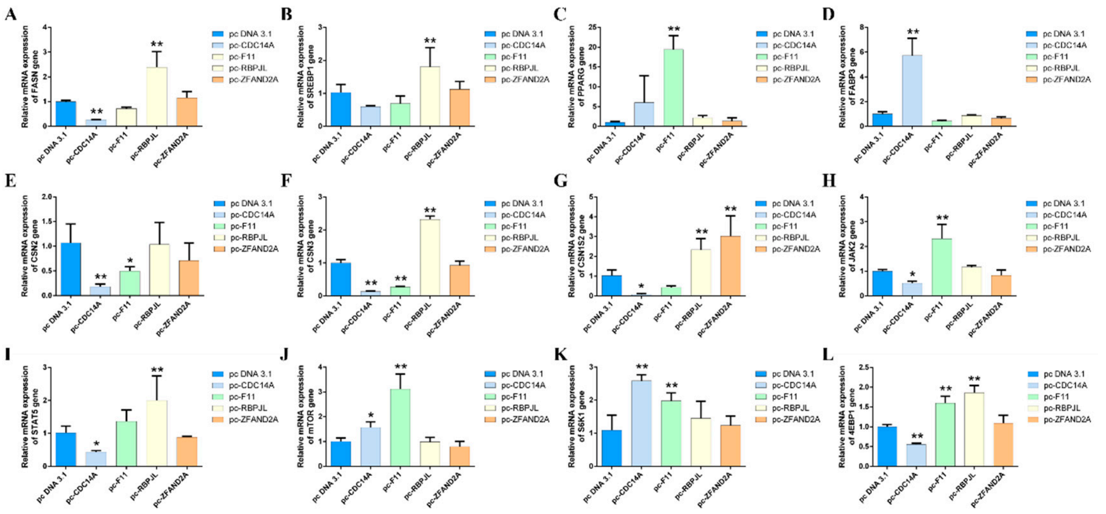

Figure S1. mRNA Expression of Lactation-Related Genes Following Overexpression of Candidate Genes.

A-D. Fold changes in mRNA levels of milk fat biosynthesis-associated genes (FASN, SREBP1, PPARG, and FABP3) upon overexpression vector treatment.

E-G. Fold changes in mRNA levels of milk protein biosynthesis-associated genes (CSN2, CSN3, and CSN1S2) upon overexpression vector treatment.

H-I. Fold changes in mRNA levels of JAK-STAT pathway-associated genes (JAK, STAT5) upon overexpression vector treatment.

J-L. Fold changes in mRNA levels of mTOR pathway-associated genes (mTOR, S6K1, 4EBP1) upon overexpression vector treatment.

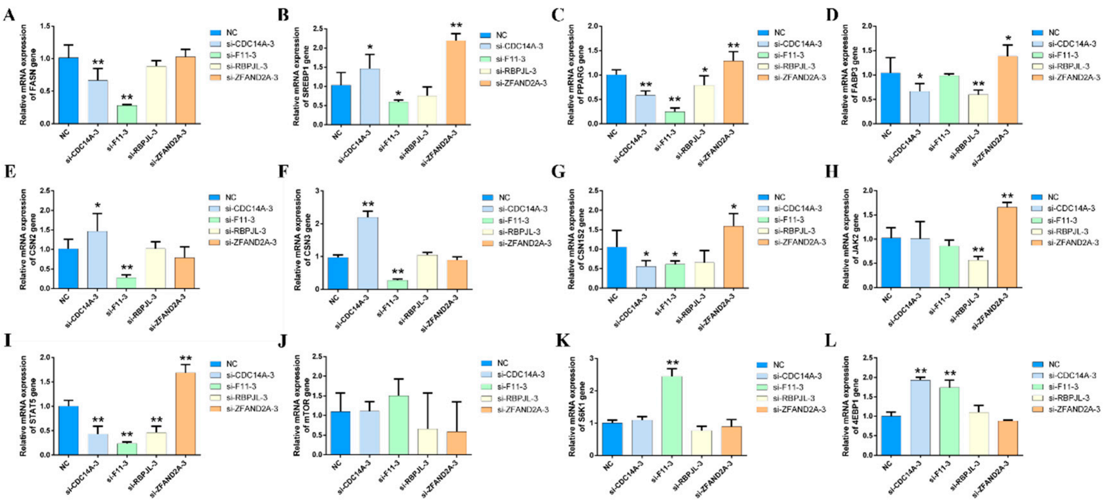

Figure S2. mRNA Expression of Lactation-Related Genes Following Silencing of Candidate Genes.

A-D. Fold changes in mRNA levels of milk fat biosynthesis-associated genes (FASN, SREBP1, PPARG, and FABP3) upon siRNA treatment.

E-G. Fold changes in mRNA levels of milk protein biosynthesis-associated genes (CSN2, CSN3, and CSN1S2) upon siRNA treatment.

H-I. Fold changes in mRNA levels of JAK-STAT pathway-associated genes (JAK, STAT5) upon siRNA treatment.

J-L. Fold changes in mRNA levels of mTOR pathway-associated genes (mTOR, S6K1, 4EBP1) upon siRNA treatment.
